# Dynamic changes of gut microbiota during progression of three Alzheimer’s disease mice models

**DOI:** 10.3389/fnins.2026.1849896

**Published:** 2026-06-23

**Authors:** Zhiyan Zou, Huan Liu, Dan Lei, Xuemin Jian, Xiaoan Li

**Affiliations:** 1 School of Life Sciences and Engineering, Southwest University of Science and Technology, Mianyang, China; 2 NHC Key Laboratory of Nuclear Technology Medical Transformation, Mianyang Central Hospital, School of Medicine, University of Electronic Science and Technology of China, Mianyang, China; 3 Department of Pediatrics, Mianyang Central Hospital, School of Medicine, University of Electronic Science and Technology of China, Mianyang, China

**Keywords:** Alzheimer’s disease, dynamic alterations, gut microbiota, microbiota-gut-brain axis, mouse models

## Abstract

**Introduction:**

Alzheimer’s disease (AD) is an age-related and progressive neurodegenerative disorder characterized by cognitive impairment and irreversible neuronal degeneration, affecting approximately 55 million individuals worldwide. Despite extensive research efforts, the underlying pathogenic mechanisms of AD remain incompletely understood, and effective therapeutic strategies for preventing or delaying disease progression are still lacking. Increasing evidence suggests that the microbiota-gut-brain axis plays an important role in neurodegenerative diseases, including AD. However, the dynamic alterations of gut microbiota during AD progression across different transgenic mouse models remain poorly characterized.

**Methods:**

In the present study, we investigated age-dependent changes in gut microbiota composition in three commonly used AD mouse models, including APP/PS1, 3xTg, and 5xFAD mice, using 16S rRNA gene sequencing. Fecal samples were collected longitudinally at 2, 4, 6, and 8 months of age to evaluate microbial diversity, community structure, and differential bacterial taxa during aging and disease progression.

**Results:**

Our results demonstrated distinct and model-dependent alterations in gut microbiota composition across different stages of AD progression. Significant changes in microbial diversity and bacterial community structure were observed among the three AD mouse models and wild-type controls. In particular, dynamic alterations in Verrucomicrobiota, Proteobacteria, and Actinobacteriota were consistently identified during aging in AD mice. In addition, β-diversity, Linear discriminant analysis effect size (LEfSe), and correlation network analyses further revealed differential microbial signatures associated with different AD mouse models and age stages.

**Discussion:**

Overall, our findings provide additional evidence that gut microbiota composition undergoes dynamic alterations during aging in multiple AD mouse models and may be associated with AD-related progression. This study may contribute to a better understanding of microbiota-associated changes during AD development and provide a basis for future mechanistic studies targeting the microbiota-gutbrain axis in AD.

## 1 Introduction

Alzheimer’s disease (AD), a common neurological disorder and also the predominant form of dementia, is characterized by the accumulation of amyloid beta (Aβ) and neurofibrillary tangles composed of hyperphosphorylated tau protein in the cortex and hippocampus regions of the brain (Khan et al., 2020; Scheltens et al., 2021; Trejo-Lopez et al., 2022). These pathological alterations ultimately lead to memory impairment, cognitive decline, psychiatric symptoms, and behavioral disturbances, thereby severely affecting patients’ daily functioning and quality of life (Maity et al., 2021). Currently, approximately 55 million people worldwide are living with AD, and this number is projected to increase to 78 million by 2030 (Alzheimers Dementia, 2024). Despite extensive research focusing on early diagnosis and the characterization of behavioral and neurological manifestations, the precise pathogenic mechanisms underlying AD remain incompletely understood (Dubois et al., 2023; Huang et al., 2024; Zhang et al., 2024). Moreover, effective therapies capable of preventing or delaying AD progression are still lacking, posing a substantial burden on global public health. Increasing evidence suggests that AD is a multifactorial disease involving complex interactions among genetic susceptibility, neuroinflammation, metabolic dysfunction, vascular abnormalities, environmental exposures, and lifestyle-related factors, rather than resulting from a single pathogenic mechanism (Calsolaro and Edison, 2016; Kivipelto et al., 2018; Livingston et al., 2020; Long and Holtzman, 2019). Among these contributing factors, increasing attention has recently been directed toward the microbiota-gut-brain axis, which may participate in the development and progression of AD through immune, metabolic, and neuroinflammatory pathways.

Recently, extensive studies have suggested that gut microbiota can influence the brain activity and function through the microbiota-gut-brain axis (Chen et al., 2022; Varesi et al., 2022; Wiatrak et al., 2022). The human gastrointestinal tract harbors approximately 1,000 microbial species and nearly 10^14^ microorganisms, which collectively play essential roles in maintaining host metabolism, immune homeostasis, and overall health (Eckburg et al., 2005; Schmidt et al., 2018). Dysbiosis of the gut microbiota, particularly the overgrowth of pathogenic microorganisms or the loss of beneficial commensals, has been implicated in the development and progression of various diseases (Adak and Khan, 2019). The composition of gut microbiota varies among individuals, and differences in microbial communities may influence host metabolism, genetic regulation, and disease susceptibility (Clemente et al., 2012). Numerous studies have demonstrated that gut microbiota participates in maintaining essential nutrient absorption, immune function, and epithelial development in the host (Belkaid and Hand, 2014; Merlo et al., 2024; Rooks and Garrett, 2016). Furthermore, the human intestine contains a complex enteric nervous system, and increasing clinical and experimental evidence suggests a close connection between gut microbiota and the central nervous system (Ma et al., 2019). Alterations in gut microbiota composition have been associated not only with gastrointestinal disorders but also with neurological diseases, including depression, anxiety, Parkinson’s disease, and AD (Bear et al., 2020; Hirayama and Ohno, 2021; Liu et al., 2019; Liu et al., 2023). Several studies have reported significant changes in gut microbiota composition and microbial diversity in both AD mouse models and patients with AD (Ferreiro et al., 2023; Liu et al., 2020). Moreover, fecal microbiota transplantation from healthy mice into AD mouse models has been shown to alleviate Aβ and tau pathology and improve cognitive function (Kim et al., 2020; Sun et al., 2019; Wang et al., 2021). Nevertheless, the dynamic alterations of gut microbiota during AD progression remain poorly understood.

Currently, Numerous animal models of AD have been developed to improve our understanding of AD pathophysiology and to facilitate the exploration of potential therapeutic strategies. In the present study, three commonly used transgenic AD mouse models, including APP/PS1, 3xTg, and 5xFAD mice, were employed, while age-matched C57BL/6 mice served as the control group. These transgenic models are widely utilized in AD research because they recapitulate several key pathological features of AD, including Aβ plaque deposition and tau-related neurodegeneration.

APP/PS1 mice co-express human amyloid precursor protein (APP) carrying the Swedish mutation and mutant presenilin-1 (PSEN1) under the control of the Thy1 promoter, resulting in elevated APP expression and progressive Aβ deposition (Lok et al., 2013). The 3xTg mouse model carries three familial AD-related mutations, including APP Swedish, MAPT P301L, and PSEN1 M146V, and develops both Aβ plaques and neurofibrillary tangles in an age-dependent manner (Pattanayak and Firdous, 2026). In addition, 5xFAD mice harbor five familial AD mutations, including three mutations in APP (Swedish K670N/M671L, Florida I716V, and London V717I) and two mutations in PSEN1 (M146L and L286V), leading to rapid and aggressive amyloid pathology (Oakley et al., 2006). Due to their distinct pathological characteristics and disease progression patterns, these three AD mouse models provide valuable tools for investigating the dynamic alterations of gut microbiota during AD progression.

In this study, three transgenic AD mouse models together with age-matched wild-type C57BL/6 mice were investigated at 2, 4, 6, and 8 months of age to characterize age-associated alterations in gut microbiota composition during the early and middle stages of AD progression using 16S rRNA gene sequencing. Considering that different AD mouse models exhibit distinct pathological trajectories and microbiota dynamics across disease development, the selected time points were designed to capture representative microbiota changes during the progression of AD-related pathology. Through comparative analysis among different AD models and control mice, this study aims to improve our understanding of the association between gut microbiota dysbiosis and AD progression, and to provide a foundation for future investigations into microbiota-related mechanisms and potential therapeutic interventions in AD.

## 2 Materials and methods

### 2.1 Animals

The APP/PS1, 3xTg, and 5xFAD transgenic mice and age-matched, C57BL6 mice (*n* = 10), were purchased from Gene & Peace Biotech Co., Ltd. (Jiangsu, China). All mice share a similar genetic background and are housed in the SPF facility at the Animal Experiment Center of Mianyang Central Hospital. Meanwhile, the mice were divided into Alzheimer’s disease (AD) groups, including APP/PS1, 3xTg, and 5xFAD groups, as well as a separate C57 group, which were kept under a 12-h light/dark cycle at 22 ± 2 °C with unrestricted access to food and water. All animal procedures were conducted in accordance with the Regulations of Experimental Animal Administration established by the State Committee of Science and Technology of the People’s Republic of China. Additionally, the experimental protocols received approval from the Animal Ethics Committee of Mianyang Central Hospital.

### 2.2 Sample collection

All stool samples were collected from the mice at 2, 4, 6, and 8 months of age. The stool samples of all mice were collected, frozen in liquid nitrogen, and then stored at −80°C for further use.

### 2.3 Bacterial 16S rRNA gene sequencing

Total bacterial genomic DNA was first extracted from fecal samples using the TIANamp Stool DNA Kit (TIANGEN Biotech, Beijing, China; Cat. No. DP328) according to the manufacturer’s instructions. Subsequently, the V3-V4 hypervariable regions of the bacterial 16S rRNA gene were amplified using the universal primers 341F (5′-CCTAYGGGRBGCASCAG-3′) and 806R (5′-GGACTACNNGGGTATCTAAT-3′), which contained unique barcode sequences for sample identification. PCR amplification was performed in a 30 μL reaction system containing 15 μL of Phusion^®^ High-Fidelity PCR Master Mix (New England Biolabs, United States), 1 μL of each primer, and approximately 10 μL of template DNA. The amplification conditions were set as follows: an initial denaturation at 98°C for 1 min, followed by 30 cycles of denaturation at 98°C for 10 s, annealing at 50°C for 30 s, extension at 72°C for 30 s, and a final extension at 72°C for 5 min. Following amplification, the PCR products were purified using AMPure XP magnetic beads (Beckman Coulter, United States) to remove primer dimers, non-specific amplification products, and small DNA fragments. Then, the purified amplicons were recovered and quantified using the Universal DNA Purification Kit (TIANGEN Biotech, Beijing, China; Cat.No.DP214-03) according to the manufacturer’s protocol prior to library construction and sequencing. Next, sequencing libraries were then constructed using the NEBNext Ultra II DNA Library Prep Kit (New England Biolabs, United States; Cat. No. E7645B) according to the manufacturer’s instructions, and the library quality was evaluated using the Agilent 2100 Bioanalyzer (Agilent Technologies, United States). Paired-end sequencing was subsequently performed on the Illumina NovaSeq6000 platform (Illumina, United States).

### 2.4 Bioinformatic analysis

Paired- end reads was assigned to samples based on their unique barcode and truncated by cutting off the barcode and primer sequence. Paired-end reads were merged using FLASH software (V1.2.11) (default parameters:–min-overlap), a very fast and accurate analysis tool, which was designed to merge paired-end reads when at least some of the reads overlap the read generated from the opposite end of the same DNA fragment, and the splicing sequences were called raw tags (Magoč and Salzberg, 2011). Subsequently, the quality filtering on the raw tags were carried out using the fastp software (V0.23.1) to obtain high-quality clean tags. To further improve sequence quality, chimeric sequences were identified and removed using the UCHIME algorithm implemented in VSEARCH software (V2.16.0) in *de novo* mode, which detects potential chimeras based on sequence abundance and internal similarity patterns. For the effective tags obtained previously, denoise was performed with DADA2 module in the QIIME2 software (V202202) to obtain initial Amplicon Sequence Variants (ASVs). Next, the species annotation was performed using QIIME2 software (V202202). The annotation database is SILVA database (release 138.1). Then, the absolute abundance of ASVs was normalized using a standard of sequence number corresponding to the sample with the least sequences. Subsequent analysis of alpha diversity and beta diversity were all performed based on the output normalized data. The top 10 taxa of each group at phylum taxonomic were selected to plot the distribution histogram of relative abundance in Perl (V5.26.2) through SVG function. Flower diagrams visually display the common and unique ASVs information among different groups, which were produced in Perl (V5.26.2) with SVG function. In order to analyze the diversity, richness and uniformity of the communities in the group, alpha diversity was calculated from 3 indices in QIIME2, including Chao1, Shannon, and Simpson. Furthermore, in order to evaluate the complexity of the community composition and compare the differences among groups, principal coordinate analysis (PCoA) was performed to get principal coordinates and visualize from complex and multidimensional data. A distance matrix of jaccard distance among groups was obtained before transformation to a new set of orthogonal axes, by which the maximum variation factor is demonstrated by first principal coordinate, and the second maximum one by the second principal coordinate, and so on. PCoA analysis was displayed by ade4 package and ggplot2 package in R software (V4.0.3). Finally, to explore the symbiotic relationship between species, spearman correlation analysis was performed across all samples to generate a species correlation coefficient matrix. The following filtering criteria were applied: (1) correlations with coefficients < 0.6 were removed; (2) self-connections between nodes were excluded; and (3) connections involving taxa with relative abundances lower than 0.005% were discarded. Based on these criteria, the microbial interaction 2D network diagrams were drew for visualization.

### 2.5 Statistical analysis

Statistical significance of beta diversity differences was assessed using permutational multivariate analysis of variance (PERMANOVA) with 999 permutations. In addition, differential taxonomic abundance among groups was analyzed using the Wilcoxon rank-sum test for pairwise comparisons and the Kruskal-Wallis test followed by Benjamini-Hochberg false discovery rate (FDR) correction for multiple-group comparisons. A *p* < 0.05 was considered statistically significant.

## 3 Results

### 3.1 The pathological characteristics of three AD mouse models at different time points

**FIGURE 1:**
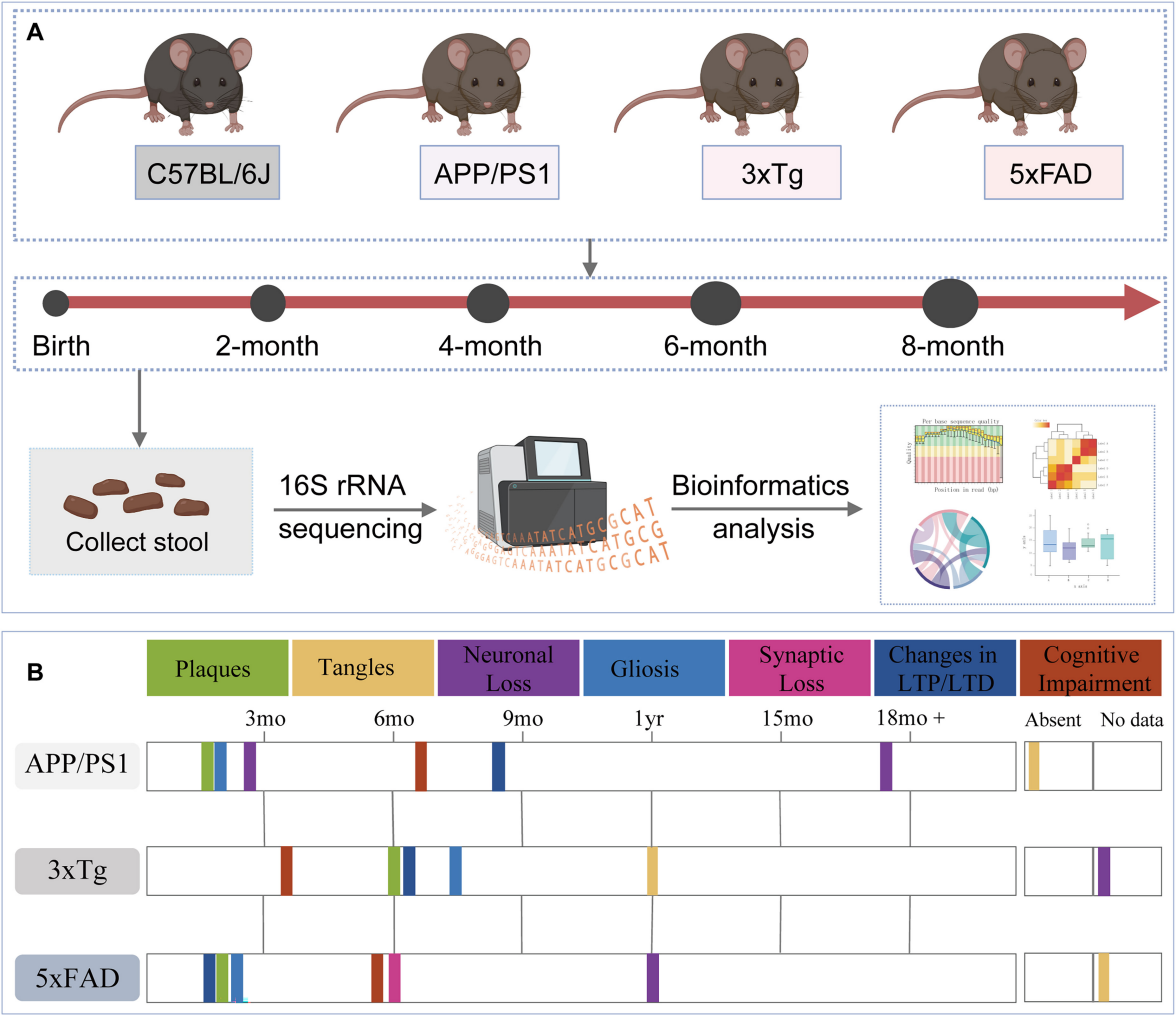
Experimental design and pathological characteristics of AD mouse models. **(A)** Schematic overview of the experimental design. Fecal samples were collected longitudinally from wild-type C57BL/6 mice and three transgenic AD mouse models (APP/PS1, 3xTg, and 5xFAD) at 2, 4, 6, and 8 months of age for gut microbiota analysis by 16S rRNA gene sequencing. **(B)** Representative pathological characteristics and disease progression features of the three AD mouse models at different age stages, including amyloid-β (Aβ) deposition and tau-related pathological alterations reported in previous studies.

To investigate age-associated alterations in gut microbiota composition in different AD mouse models, fecal samples were collected from APP/PS1, 3xTg, 5xFAD, and age-matched wild-type C57BL/6 mice at 2, 4, 6, and 8 months of age, as illustrated in Figure 1A. Figure 1B schematically summarizes the reported pathological characteristics of the three transgenic AD mouse models during disease progression, including amyloid-beta (Aβ) deposition and tau-related neuropathological alterations based on previously published studies. Although these AD mouse models share several common pathological features, the onset and progression of AD-related pathology differ among models and across time points.

### 3.2 Dynamic alterations of gut microbiota composition across different AD mouse models and age stages

**FIGURE 2:**
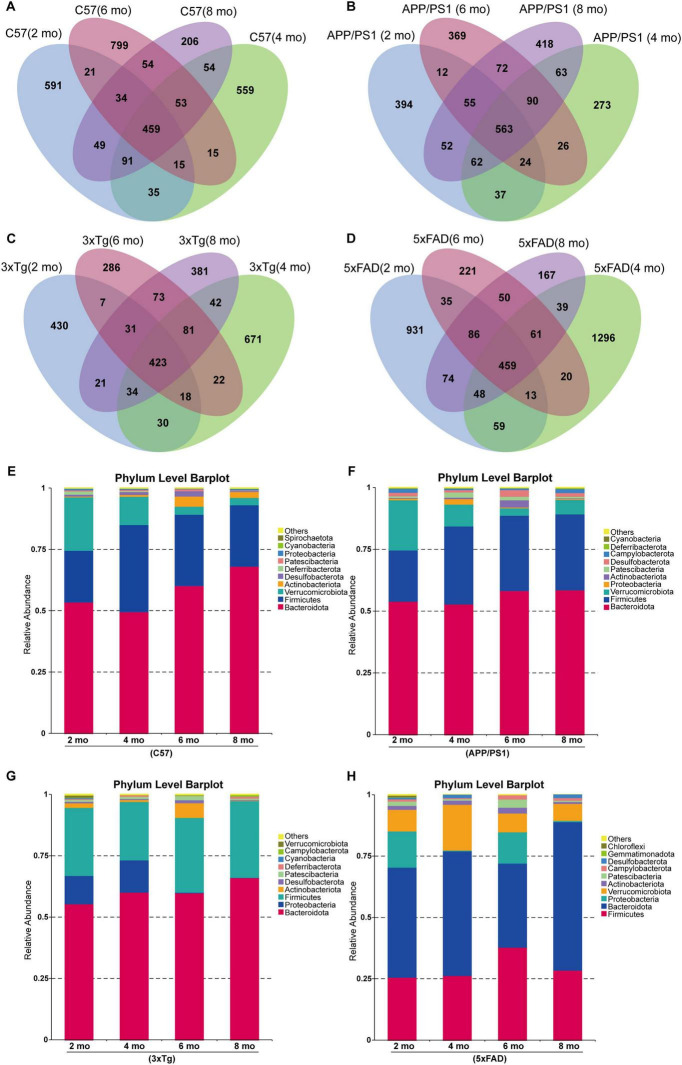
Dynamic alterations in gut microbiota composition during aging in different AD mouse models. **(A–D)** Flower diagrams showing the shared and unique ASVs among fecal samples collected at 2, 4, 6, and 8 months of age within the C57BL/6, APP/PS1, 3xTg, and 5xFAD mouse groups, respectively. **(E–H)** Relative abundance and phylum-level composition of gut microbiota in C57BL/6, APP/PS1, 3xTg, and 5xFAD mice at 2, 4, 6, and 8 months of age, respectively.

To investigate age-associated alterations in gut microbiota composition among different AD mouse models, ASV-based microbial profiling was performed in APP/PS1, 3xTg, 5xFAD, and age-matched C57BL/6 mice at 2, 4, 6, and 8 months of age. Comprehensive ASV abundance data across different taxonomic levels for all individual mouse samples are provided in Supplementary Table 1. First, shared ASVs within each mouse model across different age stages were analyzed. The C57BL/6 group exhibited 459 common ASVs across all four time points (Figure 2A). Similarly, 563, 423, and 459 common ASVs were identified in APP/PS1, 3xTg, and 5xFAD mice, respectively (Figures 2B–D), suggesting both conserved and age-dependent microbial alterations within each model.

Subsequently, phylum-level microbial composition was analyzed longitudinally within each mouse model. In C57BL/6 mice, Bacteroidota, Firmicutes, and Verrucomicrobiota were the predominant phyla at 2 and 4 months of age. However, the relative abundance of Verrucomicrobiota gradually decreased at 6 and 8 months, whereas Actinobacteriota exhibited an increased abundance during later stages (Figure 2E). In APP/PS1 mice, Bacteroidota, Firmicutes, and Verrucomicrobiota also represented the dominant phyla throughout the examined age stages. Notably, Verrucomicrobiota progressively declined with aging, while Proteobacteria and Actinobacteriota showed increased abundance at 4 and 6 months, respectively (Figure 2F). In 3xTg mice, Bacteroidota, Proteobacteria, and Firmicutes were the predominant phyla at 2 and 4 months of age. In addition, the relative abundance of Proteobacteria decreased at 4 and 6 months, whereas Actinobacteriota exhibited enrichment at 6 months (Figure 2G). Similarly, Firmicutes, Bacteroidota, and Verrucomicrobiota were the dominant phyla in 5xFAD mice across different age stages. A reduced abundance of Proteobacteria was observed at 4 and 8 months, whereas Actinobacteriota showed a modest increase at 6 months (Figure 2H).

**FIGURE 3:**
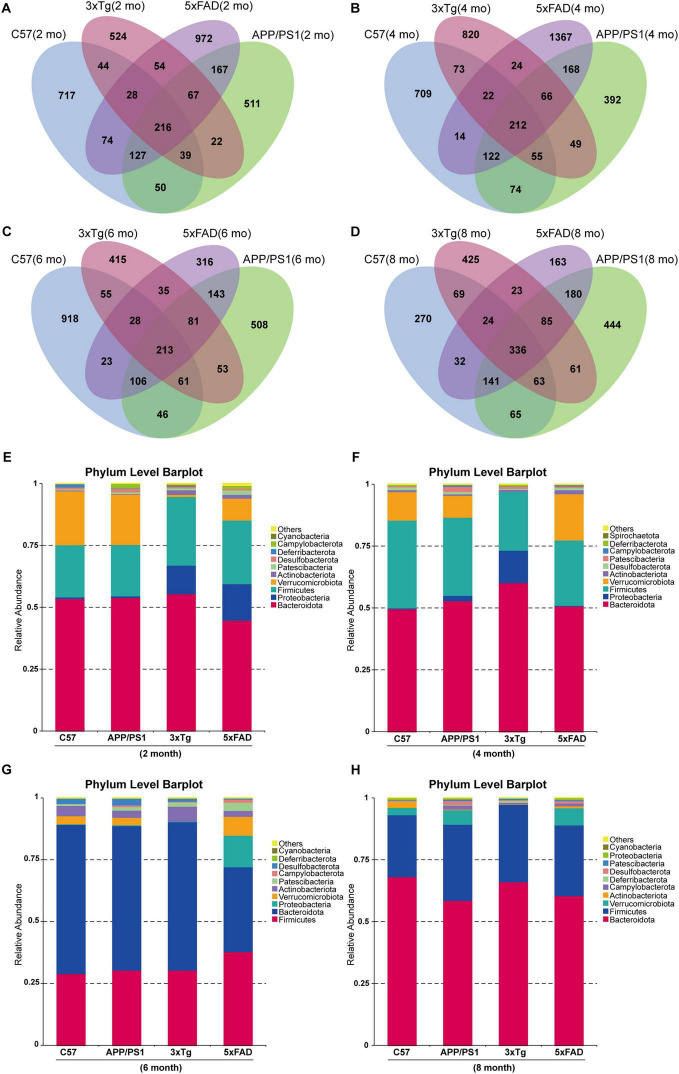
Comparative analysis of gut microbiota composition among different AD mouse models at the same age stages. **(A–D)** Flower diagrams showing the shared and unique ASVs among C57BL/6, APP/PS1, 3xTg, and 5xFAD mice at 2, 4, 6, and 8 months of age, respectively. **(E–H)** Relative abundance of the top 10 dominant gut microbial taxa at the phylum level among C57BL/6, APP/PS1, 3xTg, and 5xFAD mice at 2, 4, 6, and 8 months of age, respectively, illustrating differences in gut microbiota composition among mouse models at the same age stage.

To further compare microbial composition among different mouse models at the same age, shared ASVs were analyzed across groups. The numbers of common ASVs among the four mouse groups at 2, 4, 6, and 8 months were 216, 212, 213, and 336, respectively (Figures 3A–D). At 2 months of age, Bacteroidota, Firmicutes, and Verrucomicrobiota were the predominant phyla in C57BL/6 and APP/PS1 mice, whereas Bacteroidota, Proteobacteria, and Firmicutes were dominant in 3xTg and 5xFAD mice. In particular, Verrucomicrobiota abundance was reduced in 3xTg mice, while Proteobacteria abundance was elevated in both 3xTg and 5xFAD mice (Figure 3E). At 4 months of age, Bacteroidota, Firmicutes, and Verrucomicrobiota remained dominant in C57BL/6, APP/PS1, and 5xFAD mice, although Verrucomicrobiota abundance was lower than that observed at 2 months. In contrast, Proteobacteria remained relatively enriched in 3xTg mice (Figure 3F). Previous studies have reported that alterations in Proteobacteria abundance are associated with neuroinflammation, intestinal barrier dysfunction, and cognitive impairment in AD-related conditions (Manfredi et al., 2025). At 6 months of age, Firmicutes and Bacteroidota were the predominant phyla across all mouse groups. Notably, Verrucomicrobiota was nearly absent in 3xTg mice, while Proteobacteria abundance was increased in 5xFAD mice (Figure 3G). Similar microbial alterations were also observed at 8 months of age, with Bacteroidota and Firmicutes remaining dominant across groups and Verrucomicrobiota remaining markedly reduced in 3xTg mice (Figure 3H). Overall, these findings demonstrate that distinct AD mouse models exhibit age-dependent alterations in gut microbiota composition. In particular, changes in Verrucomicrobiota, Proteobacteria, and Actinobacteriota were consistently observed among different AD models, suggesting a potential association between gut microbial dysbiosis and AD-related pathological progression.

### 3.3 Alterations in gut microbiota α-diversity across different AD mouse models and age stages

**FIGURE 4:**
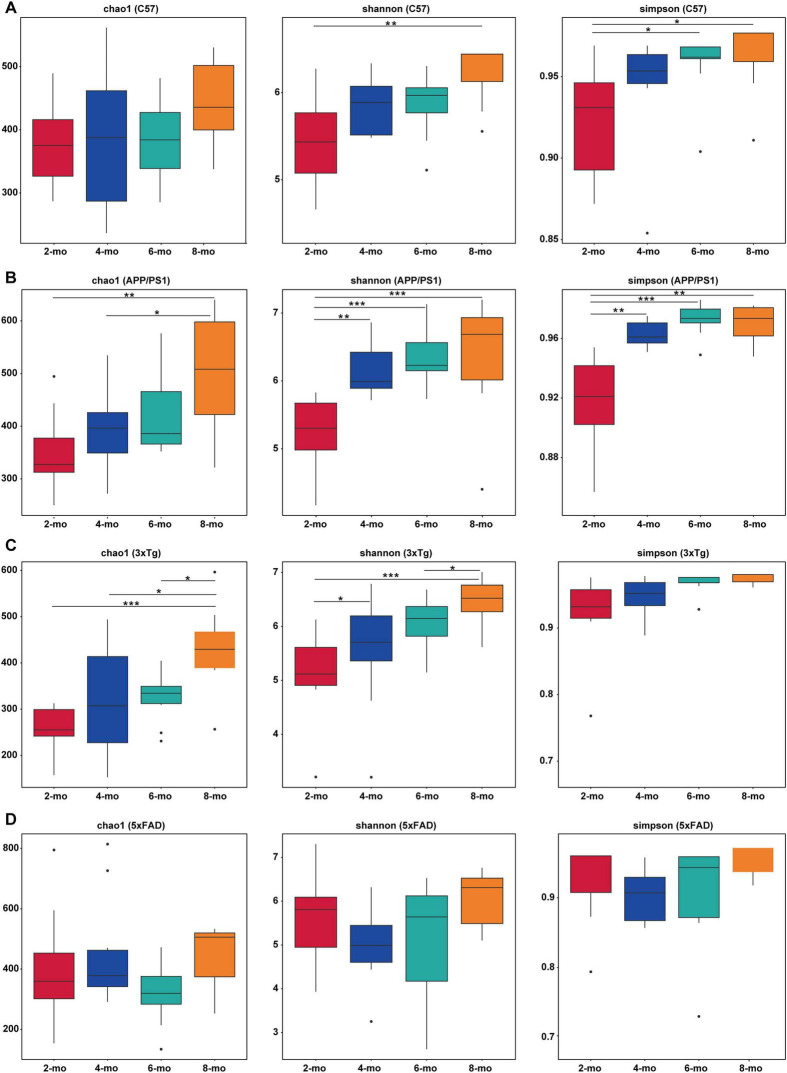
Longitudinal alterations in α-diversity of gut microbiota within each mouse model during aging. **(A–D)** α-diversity of gut microbiota in C57BL/6 **(A)**, APP/PS1 **(B)**, 3xTg **(C)**, and 5xFAD **(D)** mice at 2, 4, 6, and 8 months of age, evaluated using the Chao1, Shannon, and Simpson diversity indices. Data are presented as mean ± SEM. Statistical comparisons among different age groups within each mouse model were performed using the Kruskal–Wallis test followed by Benjamini–Hochberg false discovery rate (FDR) correction for multiple comparisons. Statistical significance was defined as *P < 0.05, **P < 0.01, and ***P < 0.001.

To further evaluate microbial richness and diversity among different mouse models and age stages, alpha diversity analysis was performed based on ASVabundance using the Chao1, Shannon, and Simpson indices. First, to investigate longitudinal alterations in microbial diversity, alpha diversity indices were compared across different age stages within each mouse model. In C57BL/6 mice, the Chao1 index did not significantly change across the four time points. However, Shannon and Simpson indices gradually increased with aging, with significant differences observed between 2 and 8 months of age (Figure 4A). Similarly, APP/PS1 mice exhibited gradual increases in Chao1, Shannon, and Simpson indices during aging. Significant differences in Chao1 index were observed among 2, 4, and 8 months of age, while Shannon and Simpson indices also demonstrated significant age-associated variations (Figure 4B). In 3xTg mice, Chao1 and Shannon indices progressively increased with aging and showed significant differences across age stages, whereas the Simpson index did not exhibit significant variation among the four time points (Figure 4C). In contrast, no significant alterations in alpha diversity indices were observed in 5xFAD mice during aging (Figure 4D).

**FIGURE 5:**
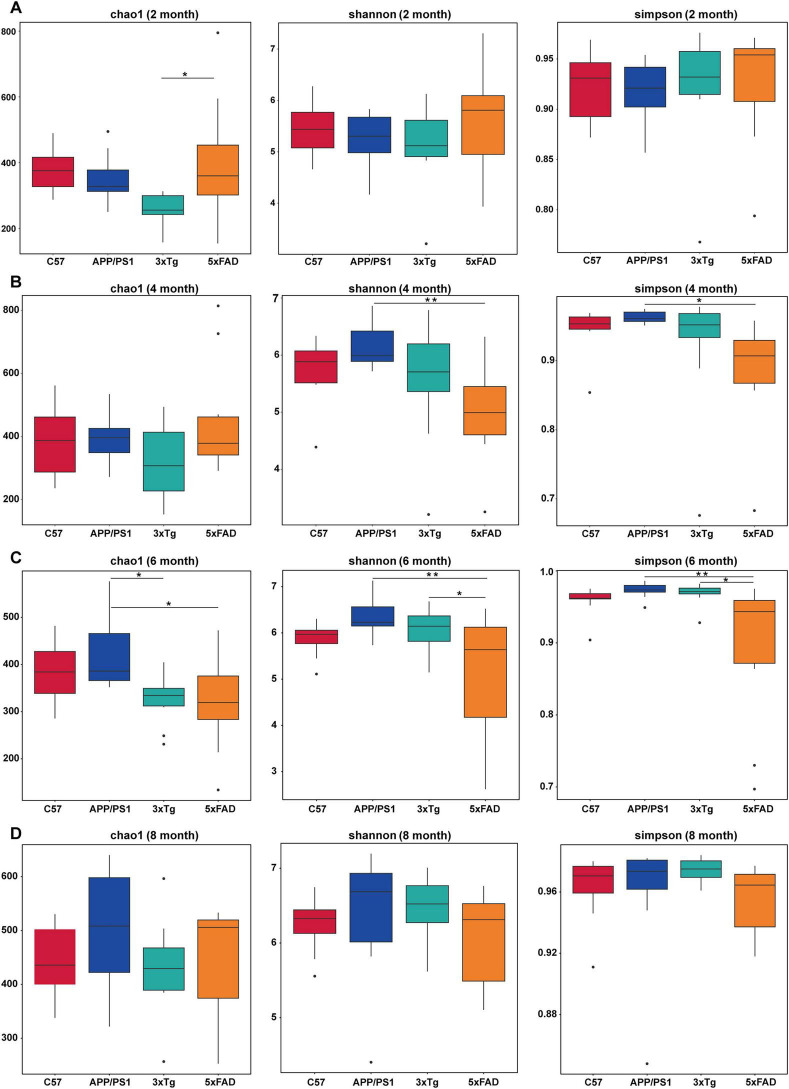
Comparative analysis of α-diversity among different mouse models at the same age stages. **(A–D)** α-Diversity of gut microbiota among C57BL/6, APP/PS1, 3xTg, and 5xFAD mice at 2 months **(A)**, 4 months **(B)**, 6 months **(C)**, and 8 months **(D)** of age, respectively, assessed using the Chao1, Shannon, and Simpson diversity indices. Data are presented as mean ± SEM. Statistical comparisons among different mouse models at the same age stage were performed using the Kruskal–Wallis test followed by Benjamini–Hochberg false discovery rate (FDR) correction for multiple comparisons. Statistical significance was defined as *P < 0.05 and **P < 0.01.

We next compared alpha diversity among different mouse models at the same age stage. At 2 months of age, the Chao1 index differed significantly between 3xTg and 5xFAD mice, whereas Shannon and Simpson indices showed no significant differences among groups (Figure 5A). At 4 months of age, Shannon and Simpson indices exhibited significant differences between APP/PS1 and 5xFAD mice, although no significant variation in Chao1 index was detected (Figure 5B). At 6 months of age, significant differences in Chao1, Shannon, and Simpson indices were observed mainly among the AD mouse models (Figure 5C). However, by 8 months of age, no significant differences in alpha diversity indices were detected among the four groups (Figure 5D). Collectively, these findings indicate that gut microbial richness and diversity exhibit dynamic and model-dependent alterations during aging in different AD mouse models.

### 3.4 Alterations in gut microbiota β-diversity across different AD mouse models and age stages

**FIGURE 6:**
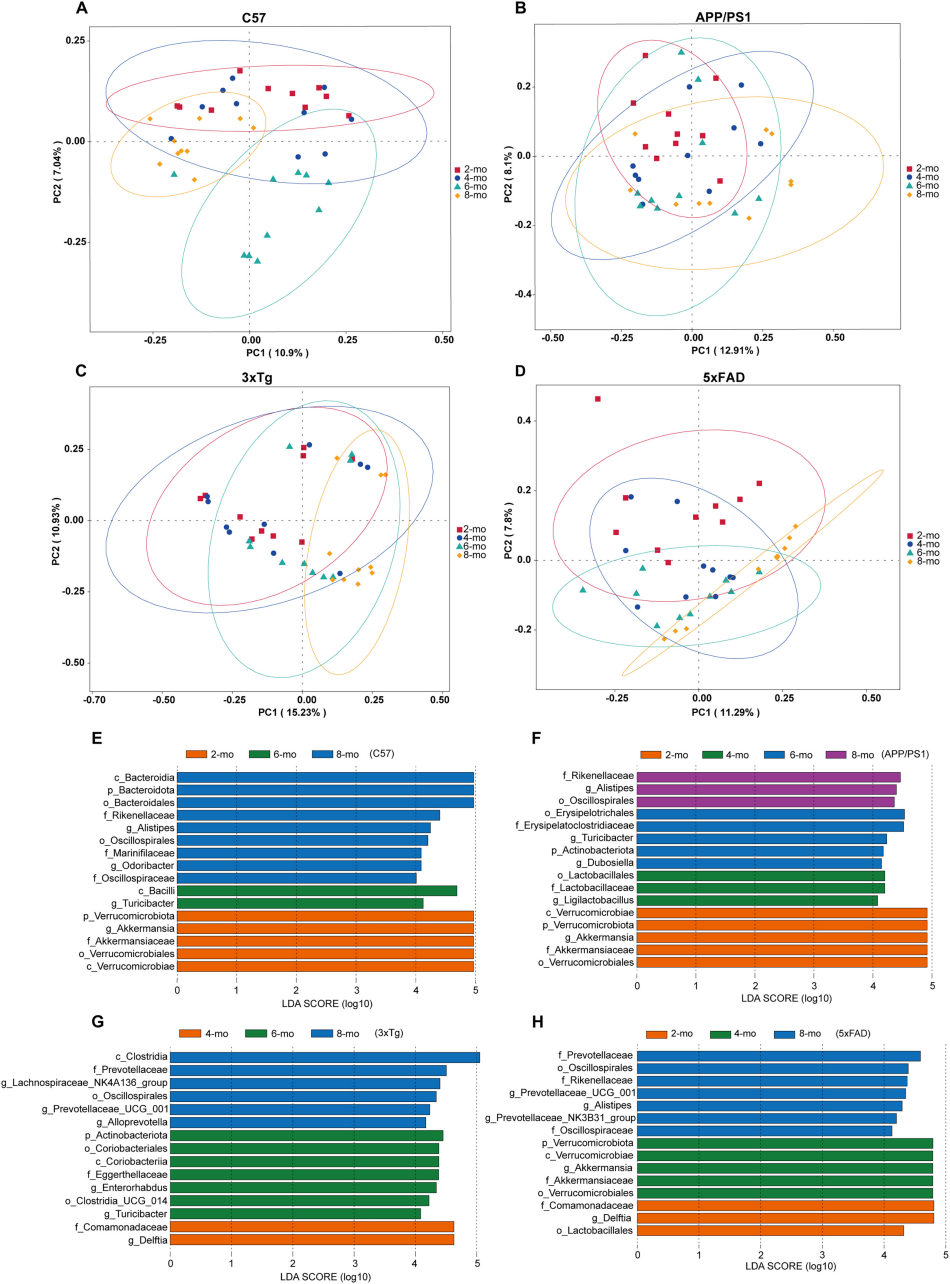
Longitudinal β-diversity and differential taxonomic analysis of gut microbiota within each mouse model during aging. PCoA analysis and taxonomic differences of gut microbiota during the progression of C57 and AD mice. **(A–D)** Principal coordinates analysis (PCoA) based on Jaccard distance showing temporal changes in gut microbial community structure within C57BL/6 **(A)**, APP/PS1 **(B)**, 3xTg **(C)**, and 5xFAD **(D)** mice across 2, 4, 6, and 8 months of age. PCoA was performed using ASV relative abundance profiles to assess β-diversity during aging. **(E–H)** Linear discriminant analysis effect size (LEfSe) analysis identifying significantly differential bacterial taxa at different age stages within C57BL/6 **(E)**, APP/PS1 **(F)**, 3xTg **(G)**, and 5xFAD **(H)** mice, respectively. Differential taxa were identified using an LDA score threshold of > 4.0 and a significance level of *P* < 0.05.

To further evaluate differences in overall microbial community composition among different mouse models and age stages, beta diversity analysis was performed using principal coordinates analysis (PCoA) based on Jaccard distance matrices. Longitudinal beta diversity analysis was conducted within each mouse model across different age stages. As shown in Figures 6A–D, samples from different age stages formed partially separated clusters in C57BL/6, APP/PS1, 3xTg, and 5xFAD mice, indicating age-associated alterations in gut microbiota composition during aging. Among these groups, 3xTg and APP/PS1 mice demonstrated relatively greater separation across time points, whereas 5xFAD mice exhibited comparatively overlapping clustering patterns.

**FIGURE 7:**
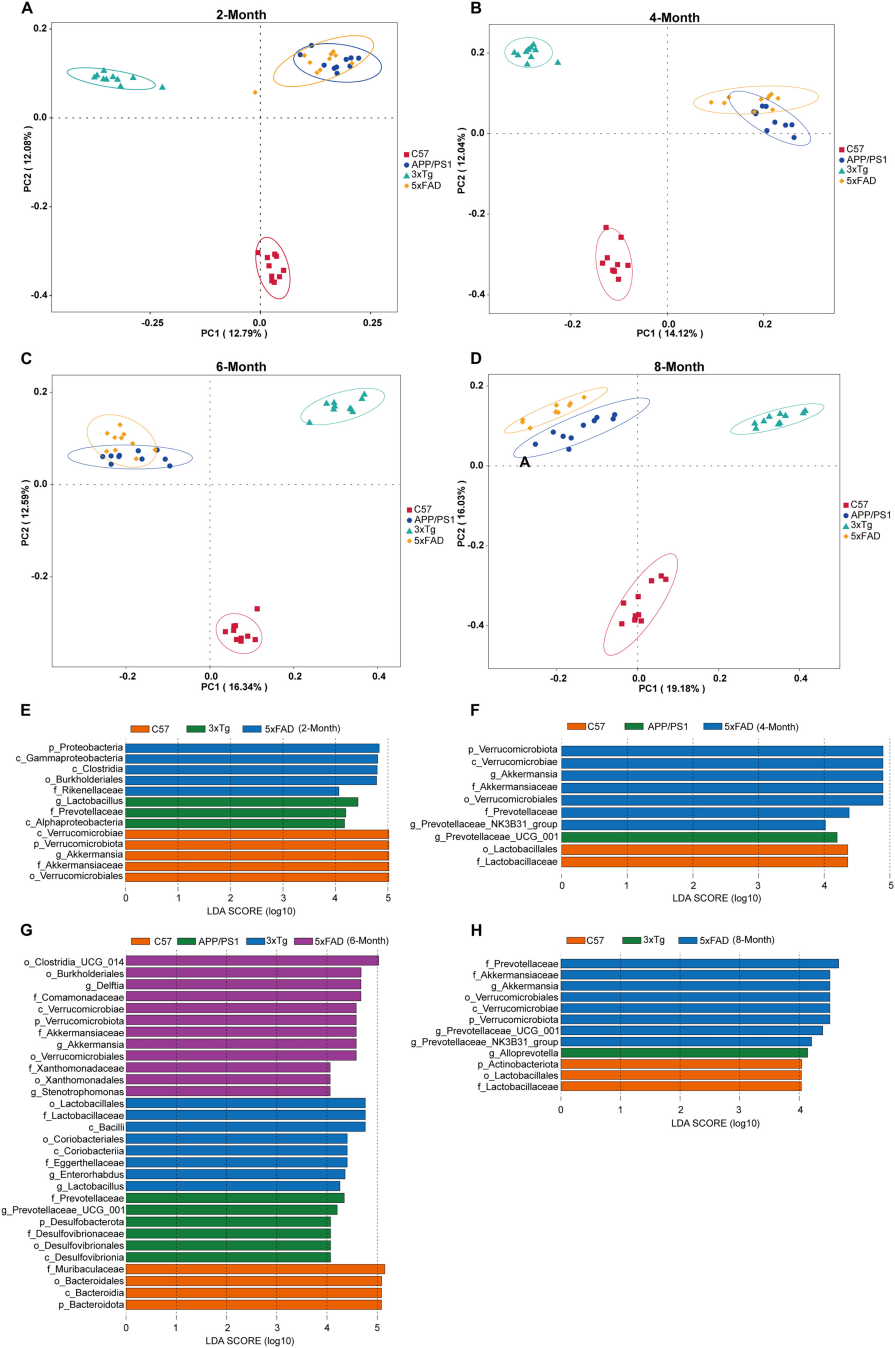
Comparative β-diversity and differential taxonomic analysis of gut microbiota among different mouse models at the same age stages. **(A–D)** Principal coordinates analysis (PCoA) based on Jaccard distance showing differences in gut microbial community structure among C57BL/6, APP/PS1, 3xTg, and 5xFAD mice at 2 months **(A)**, 4 months **(B)**, 6 months **(C)**, and 8 months **(D)** of age, respectively. PCoA was conducted using ASV relative abundance profiles to evaluate β-diversity among different mouse models at the same age stage. **(E–H)** Linear discriminant analysis effect size (LEfSe) analysis identifying significantly differential bacterial taxa among C57BL/6, APP/PS1, 3xTg, and 5xFAD mice at 2 months **(E)**, 4 months **(F)**, 6 months **(G)**, and 8 months **(H)** of age, respectively. Differential taxa were determined using an LDA score threshold of > 4.0 and a significance level of *P* < 0.05.

Furthermore, beta diversity was compared among different mouse models at the same age stage. At 2, 4, 6, and 8 months of age, C57BL/6 and 3xTg mice displayed relatively distinct clustering patterns, while APP/PS1 and 5xFAD mice showed partially overlapping microbial community distributions (Figures 7A–D). These findings suggest that different AD mouse models exhibit distinct but partially shared gut microbiota compositional characteristics during aging. Overall, the beta diversity analysis demonstrated dynamic and model-dependent alterations in gut microbial community structure across different AD mouse models and age stages.

### 3.5 Differential microbial taxa identified during aging in different AD mouse models

To further identify microbial taxa associated with aging and AD progression in different mouse models, linear discriminant analysis effect size (LEfSe) analysis was performed to detect differentially enriched bacterial taxa across age stages and mouse groups. Within individual mouse models, age-dependent alterations in differential microbial taxa were observed. In C57BL/6 mice, relatively few discriminative ASVs were identified at 2 and 4 months of age, whereas a greater number of differential taxa emerged at 8 months, suggesting increased microbial heterogeneity during aging (Figure 6E). Similarly, APP/PS1 mice exhibited dynamic alterations in differential taxa across age stages, with relatively larger numbers of enriched ASVs identified at 2 and 6 months compared with earlier time points (Figure 6F). In 3xTg mice, the number of discriminative ASVs gradually increased with aging, particularly at 6 and 8 months of age, indicating progressive alterations in microbial community structure during disease development (Figure 6G). A comparable trend was also observed in 5xFAD mice, in which relatively few differential taxa were detected at early stages, whereas substantially more enriched ASVs appeared at later stages (Figure 6H).

To further compare microbial differences among mouse models at the same age stage, LEfSe analysis was subsequently performed across the four groups. At 2, 4 and 8 months of age, relatively limited differential taxa were identified among the mouse groups (Figures 7E–G). In contrast, more pronounced microbial differences were observed at 6 months of age, with increased numbers of discriminative ASVs identified in APP/PS1, 3xTg, and 5xFAD mice (Figure 7G). By 8 months of age, the number of differential taxa decreased again, and discriminative species were mainly observed in C57BL/6, 3xTg, and 5xFAD mice (Figure 7H). Notably, the increased number of differential microbial taxa detected at 6 months of age was generally consistent with the alpha diversity analysis, which also demonstrated greater microbial diversity variation among groups at this time point. Collectively, these findings suggest that gut microbiota composition undergoes dynamic and model-dependent alterations during aging in AD mouse models, with 6 months of age representing a period of relatively pronounced microbial divergence.

### 3.6 Co-occurrence network analysis of differential gut microbial taxa during AD progression

**FIGURE 8:**
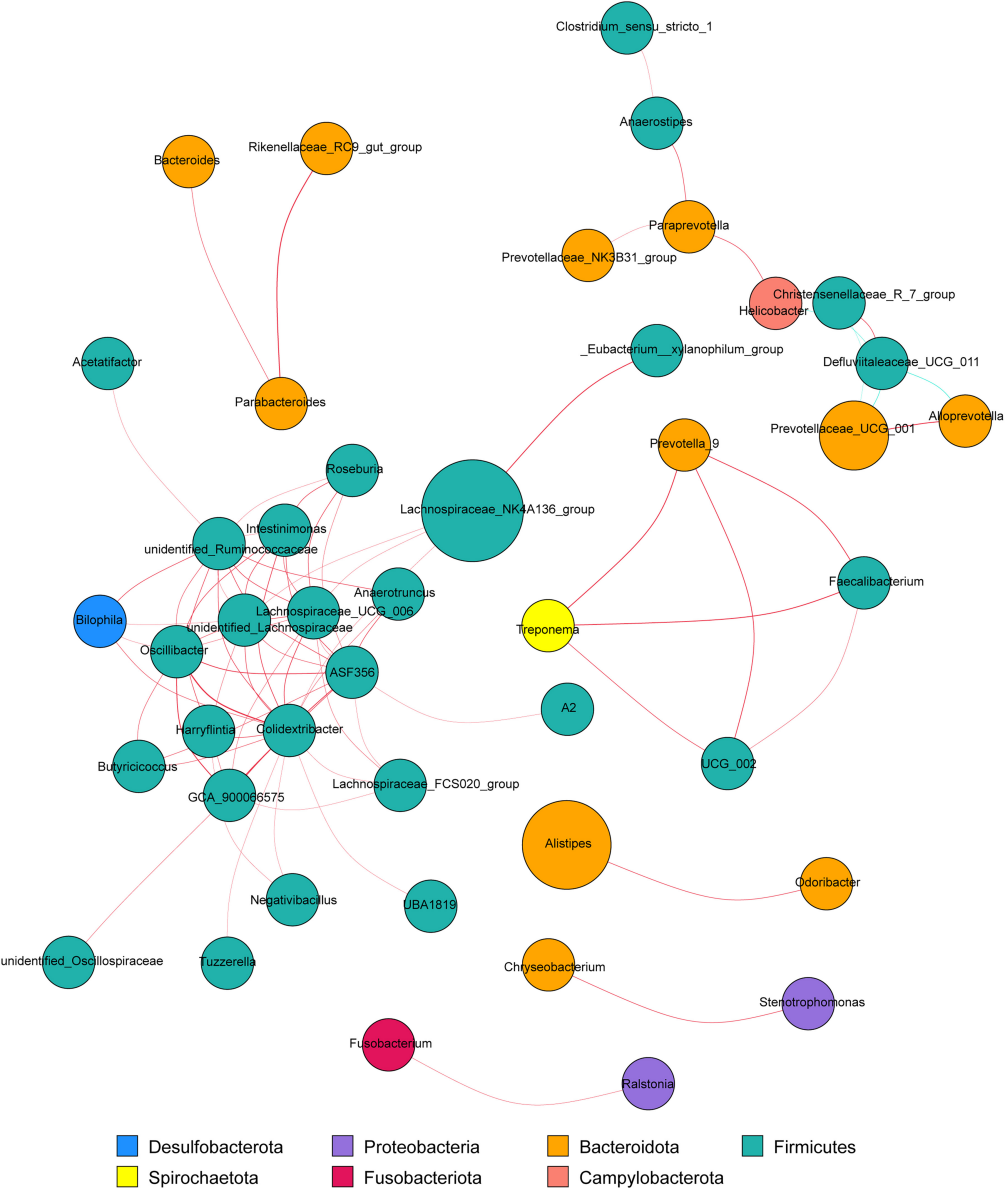
Correlation network analysis of differential gut microbial taxa during AD progression. Correlation network analysis was performed based on the relative abundance profiles of differential ASVs identified across all samples. Spearman correlation analysis was used to evaluate the relationships among dominant bacterial taxa, and only significant correlations (|*r*| > 0.6, *P* < 0.05) were included in the network visualization. In the network, each node represents an individual bacterial taxon, and the node size reflects its relative abundance. Edges indicate significant correlations between taxa, with red edges representing positive correlations and green edges representing negative correlations. The network analysis revealed potential co-occurrence and interaction patterns among gut microbial taxa associated with aging and AD-related microbial alterations.

To further investigate the potential interactions among differential gut microbial taxa during AD progression, co-occurrence network analysis was performed based on ASVrelative abundance profiles across all samples. As shown in Figure 8, the network analysis revealed several highly connected microbial taxa within the gut microbial community. Among them, Lachnospiraceae_NK4A136_group, Alistipes, and Prevotellaceae_UCG_001 exhibited relatively high abundance and strong connectivity within the co-occurrence network, suggesting that these taxa may play important roles in maintaining microbial community structure during aging and AD progression.

In addition, significant positive correlations were identified between Lachnospiraceae_NK4A136_group and Eubacterium_xylanophilum_group, as well as between Alistipes and Odoribacter, indicating potential cooperative or co-enrichment relationships among these taxa. A strong positive correlation was also observed between Prevotellaceae_UCG_001 and Alloprevotella. In contrast, Prevotellaceae_UCG_001 exhibited a negative correlation with Defluviitaleaceae_UCG_011, suggesting potential competitive or mutually exclusive relationships between these bacterial taxa. Furthermore, positive correlations were observed among Lachnospiraceae_NK4A136_group, Anaerotruncus, Lachnospiraceae_UCG_006, and unidentified_Lachnospiraceae, indicating the presence of potentially coordinated microbial communities within the Lachnospiraceae-related bacterial cluster. Overall, these findings suggest that gut microbial taxa exhibit complex co-occurrence patterns during aging and AD progression, and that specific bacterial groups may participate in coordinated microbial community alterations associated with AD-related gut dysbiosis.

## 4 Discussion

The present study systematically characterized age-associated alterations in gut microbiota composition across three widely used AD mouse models (APP/PS1, 3xTg, and 5xFAD) and age-matched C57BL/6 control mice at 2, 4, 6, and 8 months of age. Our results demonstrated dynamic and model-dependent changes in gut microbial diversity, community structure, and differential bacterial taxa during aging. Notably, several microbial alterations were more pronounced in AD mouse models than in wild-type mice, suggesting a potential association between gut microbiota dysbiosis and AD-related pathological progression.

Consistent with previous studies, the gut microbiota composition of both wild-type and AD mice changed dynamically with aging (Badal et al., 2020). In C57BL/6 mice, Bacteroidota, Firmicutes, and Verrucomicrobiota predominated during the early stages of life, whereas Verrucomicrobiota gradually decreased and Actinobacteriota increased at later time points. These findings support the concept that aging itself can influence gut microbial community structure and diversity (Li et al., 2019). Importantly, although similar microbial trends were observed in AD mouse models, the timing and magnitude of these alterations differed from those in wild-type mice. In particular, Verrucomicrobiota depletion appeared earlier and was more pronounced in 3xTg and 5xFAD mice than in C57BL/6 mice. For example, Verrucomicrobiota abundance was markedly reduced in 3xTg mice as early as 2–4 months of age, whereas a comparable decline in C57BL/6 mice mainly occurred at later stages. Previous studies have reported that Verrucomicrobiota, especially Akkermansia-related taxa, play important roles in maintaining intestinal barrier integrity and regulating host immune homeostasis (Cani and de Vos, 2017). Therefore, the earlier reduction of Verrucomicrobiota observed in AD mouse models may reflect accelerated gut microbial dysbiosis during AD progression.

In addition, increased abundance of Proteobacteria and Actinobacteriota was observed in APP/PS1, 3xTg, and 5xFAD mice during middle-to-late age stages, particularly around 4–6 months of age. Notably, these microbial alterations temporally coincided with previously reported periods of accelerated Aβ accumulation and neuroinflammatory progression in these AD mouse models (Shin et al., 2015). Proteobacteria enrichment has been widely considered a potential marker of gut microbial instability and chronic inflammation (Ajith and Sreejith, 2026). Previous studies have also demonstrated that elevated Proteobacteria abundance is associated with intestinal barrier dysfunction, systemic inflammation, and cognitive impairment in both AD patients and transgenic AD mouse models (Hung et al., 2022; Mustafa et al., 2025). Therefore, our findings suggest that the progressive expansion of Proteobacteria and Actinobacteriota may be associated with age-dependent gut microbial dysbiosis during AD progression. Moreover, the more substantial microbial alterations observed in 3xTg and 5xFAD mice compared with APP/PS1 mice may partially reflect differences in disease severity and pathological progression among these models. Previous studies have shown that 3xTg and 5xFAD mice develop earlier and more aggressive AD-related pathology than APP/PS1 mice, including accelerated amyloid deposition, synaptic dysfunction, and neuroinflammation (Oakley et al., 2006; Oddo et al., 2003). Collectively, these findings support the possibility that gut microbiota alterations may dynamically accompany disease progression in AD mouse models.

The alpha diversity indices (Shannon, Simpson, and Chao1) provided additional insights into age-associated alterations in gut microbial richness and community evenness among different mouse models. Although no significant differences in alpha diversity were observed among groups at 2 months of age, more apparent variations emerged at later stages, particularly in APP/PS1 and 3xTg mice. These findings suggest that gut microbial diversity undergoes dynamic alterations during aging and AD progression. Previous studies have similarly reported that aging and neurodegenerative disorders are frequently accompanied by reduced microbial stability and altered gut microbial diversity (Claesson et al., 2012; Dinan and Cryan, 2017). In addition, beta diversity analysis based on principal coordinates analysis (PCoA) demonstrated distinct clustering patterns among the different mouse groups at multiple age stages, indicating differences in overall microbial community structure between AD mouse models and wild-type controls. Notably, microbial community separation became more apparent in APP/PS1 and 3xTg mice during middle-to-late stages, suggesting progressive divergence of gut microbiota composition during AD-related pathological progression.

Several bacterial taxa identified in this study may be associated with gut microbial dysbiosis during AD progression. For example, Verrucomicrobiota, which includes beneficial mucin-degrading bacteria such as Akkermansia, has been reported to contribute to intestinal barrier maintenance and immune regulation (Ma et al., 2025). In the present study, Verrucomicrobiota abundance progressively decreased in AD mouse models, particularly in 3xTg mice at earlier stages, suggesting impaired microbial homeostasis during disease progression. Conversely, Proteobacteria abundance increased in several AD mouse models during aging. Previous studies have proposed that expansion of Proteobacteria may represent a microbial signature of intestinal dysbiosis and chronic inflammation (Shin et al., 2015). Elevated Proteobacteria abundance has also been associated with increased intestinal permeability and systemic inflammatory responses in neurodegenerative diseases (Ajith and Sreejith, 2026). Similarly, increased Actinobacteriota abundance observed in APP/PS1 and 3xTg mice may reflect altered microbial metabolic activity and immune regulation during AD progression. Furthermore, co-occurrence network analysis revealed strong associations among several microbial taxa, including Lachnospiraceae_NK4A136_group, Alistipes, and Prevotellaceae_UCG_001. Previous studies have suggested that these bacterial taxa may participate in short-chain fatty acid metabolism, immune modulation, and gut-brain axis communication (Dalile et al., 2019; Nagpal et al., 2019). Therefore, the coordinated alterations observed in these microbial taxa may reflect dynamic microbial community remodeling during AD progression. Overall, our findings support the concept that gut microbiota composition undergoes dynamic and model-dependent alterations during aging in AD mouse models. However, because behavioral and pathological assessments were not performed in the present study, the direct relationship between gut microbiota dysbiosis and cognitive impairment or AD pathology requires further investigation.

Overall, the present study demonstrated that gut microbiota composition undergoes dynamic and model-dependent alterations during aging in different AD mouse models. Significant changes in microbial diversity, community structure, and differential bacterial taxa were observed across APP/PS1, 3xTg, and 5xFAD mice at different age stages, suggesting a potential association between gut microbial dysbiosis and AD-related pathological progression. Previous studies have increasingly suggested that the microbiota-gut-brain axis may participate in neuroinflammatory regulation, immune homeostasis, and metabolic signaling during neurodegenerative diseases, including AD (Ma et al., 2025; Wang et al., 2023). In particular, alterations in bacterial taxa such as Verrucomicrobiota, Proteobacteria, and Lachnospiraceae-related genera have been reported in both AD patients and transgenic AD mouse models (Dalile et al., 2019; Shin et al., 2015; Vacca et al., 2020). Consistent with these findings, our study identified dynamic alterations in these microbial taxa across different AD mouse models and aging stages.

However, it is important to emphasize that the present study primarily provides observational evidence regarding gut microbiota alterations during AD progression. Although the identified microbial changes were temporally associated with aging and disease-related stages in AD mouse models, the current data do not establish a direct causal relationship between gut microbiota dysbiosis and AD pathology. Therefore, it remains unclear whether these microbial alterations contribute to disease progression or represent secondary changes associated with aging, systemic inflammation, metabolic dysfunction, or other disease-related factors. Several limitations of this study should also be acknowledged. First, behavioral assessments and neuropathological validation experiments, including cognitive testing and histopathological analysis of Aβ and phosphorylated tau deposition, were not performed in the present study. As a result, direct correlations between gut microbiota alterations and AD-related pathological severity could not be established. Second, the study relied on 16S rRNA amplicon sequencing, which primarily provides taxonomic information but lacks functional and metabolic characterization of the gut microbiota. Third, mechanistic validation experiments, such as fecal microbiota transplantation, germ-free animal colonization, antibiotic depletion, or targeted probiotic intervention studies, were not conducted. Therefore, the functional roles of specific microbial taxa in AD progression remain to be further investigated. Future studies integrating longitudinal behavioral assessments, neuropathological analyses, metagenomic or metabolomic profiling, and microbiota-targeted intervention experiments will be necessary to clarify the mechanistic relationships between gut microbiota dysbiosis and AD progression. Nevertheless, the present findings provide additional evidence supporting dynamic alterations of the gut microbiota during aging in multiple AD mouse models and may contribute to a better understanding of microbiota-associated changes during AD progression.

## 5 Conclusion

In conclusion, the present study demonstrated that gut microbiota composition undergoes dynamic and model-dependent alterations during aging in APP/PS1, 3xTg, and 5xFAD AD mouse models. Significant changes in microbial diversity and differential bacterial taxa, including Verrucomicrobiota, Proteobacteria, and Actinobacteriota, were identified across different age stages. These findings provide additional evidence supporting an association between gut microbiota dysbiosis and AD-related progression. Although the underlying mechanisms require further investigation, this study may contribute to a better understanding of microbiota alterations during AD progression and provide a basis for future microbiota-targeted studies in AD.

## Data Availability

The raw 16S rRNA sequencing datasets generated and analyzed during the current study have been deposited in the NCBI BioProject database under accession number PRJNA1464335. The datasets are publicly available at: https://www.ncbi.nlm.nih.gov/bioproject/PRJNA1464335.
